# Anxiety, depression, traumatic stress and COVID-19-related anxiety in the UK general population during the COVID-19 pandemic

**DOI:** 10.1192/bjo.2020.109

**Published:** 2020-10-10

**Authors:** Mark Shevlin, Orla McBride, Jamie Murphy, Jilly Gibson Miller, Todd K. Hartman, Liat Levita, Liam Mason, Anton P. Martinez, Ryan McKay, Thomas V. A. Stocks, Kate M. Bennett, Philip Hyland, Thanos Karatzias, Richard P. Bentall

**Affiliations:** Ulster University, Northern Ireland; Ulster University, Northern Ireland; Ulster University, Northern Ireland; University of Sheffield, England; University of Sheffield, England; University of Sheffield, England; University College London, England; University of Sheffield, England; Royal Holloway, University of London, England; University of Sheffield, England; Liverpool University, England; Maynooth University, Ireland; Edinburgh Napier University, Scotland; University of Sheffield and Liverpool University, England

**Keywords:** COVID-19 pandemic, anxiety, depression, traumatic stress, UK general population survey

## Abstract

**Background:**

The COVID-19 pandemic has created an unprecedented global crisis, necessitating drastic changes to living conditions, social life, personal freedom and economic activity. No study has yet examined the presence of psychiatric symptoms in the UK population under similar conditions.

**Aims:**

We investigated the prevalence of COVID-19-related anxiety, generalised anxiety, depression and trauma symptoms in the UK population during an early phase of the pandemic, and estimated associations with variables likely to influence these symptoms.

**Method:**

Between 23 and 28 March 2020, a quota sample of 2025 UK adults aged 18 years and older, stratified by age, gender and household income, was recruited by online survey company Qualtrics. Participants completed standardised measures of depression, generalised anxiety and trauma symptoms relating to the pandemic. Bivariate and multivariate associations were calculated for demographic and health-related variables.

**Results:**

Higher levels of anxiety, depression and trauma symptoms were reported compared with previous population studies, but not dramatically so. Anxiety or depression and trauma symptoms were predicted by young age, presence of children in the home, and high estimates of personal risk. Anxiety and depression were also predicted by low income, loss of income and pre-existing health conditions in self and others. Specific anxiety about COVID-19 was greater in older participants.

**Conclusions:**

This study showed a modest increase in the prevalence of mental health problems in the early stages of the pandemic, and these problems were predicted by several specific COVID-related variables. Further similar surveys, particularly of those with children at home, are required as the pandemic progresses.

Severe acute respiratory syndrome coronavirus 2 (SARS-CoV-2) was first detected in Wuhan, China, on 31 December 2019. The disease it causes has been named COVID-19. The first UK coronavirus case was confirmed on 31 January 2020, and on 11 March 2020 the World Health Organization declared the global spread of COVID-19 to be a pandemic. Since then there have been rapidly increasing cases and deaths associated with the virus globally and in the UK. On the evening of 23 March 2020, the UK Prime Minister announced extensive restrictions on freedom of movement, the closure of non-essential businesses and the requirement to stay at home except for limited purposes. The mental health consequences for the population of an existential threat on the scale of the current pandemic, and of the associated restrictions on movement and social gatherings, are not well understood. There has been research on the psychological effects of other infectious respiratory diseases (IRDs) such as SARS, the H1N1 flu pandemic and MERS. However, with a few exceptions, which are mostly from the far east and have focused largely on anxiety and its influence on risk perception and health behaviours rather than mental health more broadly,^1,2^ these studies have predominantly considered healthcare workers^3,4^ and patients.^5^ This absence of knowledge is troubling because there is plausible evidence from modelling that emotional and behavioural responses to a pandemic may affect its course,^6^ and because the burden of population mental ill-health may have implications for resources during the pandemic and national recovery afterwards. In 2003, the Canadian National Advisory Committee on SARS and Public Health,^7^ proposed that a ‘systemic perspective’, which focused not only on medical staff and patients but also on the general population, should be prioritised by all those engaged in IRD psychosocial research. A similar approach was advocated in a recent UK expert panel convened by the Academy of Medical Sciences and the mental health research charity MQ.^8^

Here, we report initial findings from the first wave of a longitudinal, multi-wave survey of the social and psychological effects of COVID-19 on the UK population, conducted by researchers in seven UK and Irish universities (the COVID-19 Psychological Research Consortium).^9^ Of note, in a mirror study with similar methodology, we recently reported the social and psychological effects of COVID-19 on the population of the Republic of Ireland.^10^ The primary aim of this study was to assess the levels of anxiety, depression and traumatic stress, based on validated self-report measures, in a large, representative community sample during an early stage of the pandemic, between 23 and 28 March 2020. Based on the scant previous studies^11^ and given the dramatic restrictions imposed because of COVID-19, we expected higher levels of common psychological and stress symptoms compared with previous population estimates. Our secondary aim was to identify groups that are psychologically vulnerable during the pandemic, by assessing the relationship between levels of anxiety, depression and traumatic stress and (a) age; (b) household income; (c) economic threat due to COVID-19; (d) health-related risk factors (being male, self or close friend or relative having a pre-existing serious health condition); (e) COVID-19 infection status; (f) anxiety specifically related to COVID-19; (g) perceived risk of COVID-19 infection; (h) living in an urban area; (i) living as a lone adult and (j) living with children in the home.

## Method

### Recruitment and participants

Data collection started on 23 March 2020, 52 days after the first confirmed COVID-19 case in the UK and on the same day that the UK Prime Minister announced at 8.30 pm the ‘lockdown’ that required all people in the UK to stay at home except for very limited purposes, and was completed on 28 March 2020. The fieldwork was conducted by the survey company Qualtrics. The UK adult population aged 18 years and older was the target population, and quota sampling methods were used to ensure that the sample was representative of this population in terms of age and gender, based on 2016 population estimates from Eurostat, and household income based on the 2017 Office for National Statistics household income bands. Qualtrics provides an online platform to securely house data and leverages partners to connect with potential participants who could have been alerted to the study in one of two ways: (a) they opted to enter studies they were eligible for themselves by signing up to a panel platform; or (b) they received automatic notification through a partner router which alerted them to studies for which they were eligible (via email, SMS or in-app notifications). Importantly, to avoid self-selection bias, survey invitations to eligible participants only provided general information and did not include specific details about the contents of the survey. Participants were required to be an adult (aged 18 years or older), able to read and write in English, and a resident of the UK. No other exclusion criteria were applied. Panel members were not obliged to take part in the study.

For purposes of quota sampling for age, gender and household income, Qualtrics proceeded as follows during the 6 days of fieldwork: (a) respondents in ‘hard to reach’ quota groups (e.g. young adults in the highest income bands) were prioritised and targeted first; (b) next, the focus shifted to allow the quotas to ‘fill up’ naturally, without specific targeting; and (c) finally, a switch back to targeting respondents to fill incomplete quotas ensued. Participants followed a link to a secure website and completed all surveys online. The invite link was active for a participant until a quota they would have qualified for was reached but after the quota was filled; previously eligible respondents were prevented from taking part in this study. Participants were informed about the purpose of the study, that their data would be treated in confidence, that geolocation would be used to determine the area in which they lived, and of the right to terminate the study at any time without giving a reason. All participants provided informed consent prior to completing the survey and were directed to contact the National Health Service 111 COVID-19 helpline at the end of the survey if they experienced any distress or had additional concerns about COVID-19. Ethical approval for the study was granted by the ethical review board of Sheffield University (the reference number for ethical approval is 033759).

Qualtrics employed checks to identify and remove potential duplicate respondents or any participants who completed the survey in less than the minimum completion time (half the median time of the ‘soft-launch’ with 50 participants) to ensure responses were trustworthy. The pre-recruitment quotas were achieved with a high level of accuracy; the quotas were obtained to within 1 % for gender, 0.1–0.6 % for age bands and 0.25–1 % for household income bands. The 2014 Adult Psychiatric Morbidity Survey in England estimated the rate of post-traumatic stress disorder (PTSD) to be 4.4 %;^12^ this was lower than the rates for anxiety and depression. To detect a disorder with a prevalence of 4 %, with precision of 1 % and a 95 % confidence level, a sample size of 1476 was required. However, estimating the prevalence of disorders with a low prevalence (<5 %) may result in a small number of ‘cases’ being identified. For instance, a sample size of 1476 and prevalence of 4 % will identify approximately 60 cases and, if follow-up analyses are based only on these cases, tests may be underpowered. To detect a correlation of 0.30, with alpha = 0.05 and power of 0.80, 84 cases are required (or an overall sample size of 2100). As a compromise between ensuring adequate sampling to reliably estimate prevalence and adequate power for subgroup analysis, a target sample size of 2000 participants was set.

Given the dual processes used by Qualtrics and partners to recruit respondents to quotas, it was not possible to determine the number of survey invitations that were distributed to panel members, or indeed the number of panellists who were alerted to the survey and who did or did not complete the survey (i.e. the response rate). Qualtrics did provide some metrics for the study, as follows: 159 respondents did not provide full informed consent and were screened out; 35 respondents who completed the survey from outside the UK or were aged under 18 years were also screened out; and, to ensure responses were trustworthy, 77 participants who completed the survey in less than the minimum completion time were removed, as were 64 potential duplicate respondents. This resulted in a sample of 2025 participants who completed the survey over 6 days of fieldwork.

Subsequent checks ensured that the participants were also representative of the population in terms of voting history, number of people in household and other important demographic characteristics.^9^

Participants were recruited from the four countries of the UK, proportional to their relative population sizes: England (86.9 %), Wales (3.1 %), Scotland (7.8 %), Northern Ireland (2.3 %). The mean age of the sample was 45.44 years (median = 45.00, s.d. = 15.90, range 18–83), and 51.7 % (*n* = 1047) were female, 48.0 % were male (*n* = 972) and 0.3 % (*n* = 6) checked the transgender/prefer not to say/other option. Most reported that they were born in the UK (90.6 %, *n* = 1834) and grew up (spent most of their life up to 16 years of age) in the UK (92.4 %, *n* = 1872). Participants reported their ethnicity as follows: White British/Irish (*n* = 1732, 85.5 %), White non-British/Irish (*n* = 116, 5.7 %), Indian (*n* = 41, 2.0 %), Pakistani (*n* = 27, 1.3 %), Chinese (*n* = 19, 0.9 %), other Asian/African–Caribbean/African/Arab/Bangladeshi/Other (*n* = 90, 4.30 %). Regarding participants’ highest level of educational achievement, 19.0 % (*n* = 385) had completed O-Level/GCSE or similar, 18.1 % (*n* = 366) had completed A-Level or similar, 28.2 % (*n* = 572) had completed an undergraduate degree and 15.6 % (*n* = 316) had completed a postgraduate degree, with 19.1 % (*n* = 386) reporting no qualifications, diploma, other qualifications or technical qualification. Nearly half of the respondents were in full-time employment (48.8 %, *n* = 988), 15.0 % (*n* = 303) were in part-time employment, 16.5 % (*n* = 334) were retired, 4.7 % (*n* = 95) were students, 5.1 % (*n* = 103) were currently unemployed and seeking work, 3.4 % (*n* = 69) were not working owing to disability, and 6.6 % (*n* = 133) were unemployed and not seeking work.

### Measures

#### Demographic

Self-reported gender and age were recorded, and age was also categorised into a six-level variable for the regression analysis.

#### Living area

Participants were asked ‘Do you consider yourself to live in:’ and were required to choose one of the options provided: ‘City’, ‘Suburb’, ‘Town’ or ‘Rural’.

Lone adult: Participants were asked ‘How many adults (18 years or above) live in your household (including yourself)?’ and were provided with options ranging from ‘1’ to ‘10 or more’. The data were recoded into a binary variable to represent living alone.

#### Children

Participants were asked ‘How many children (below the age of 18) live in your household?’ and were provided with options ranging from ‘1’ to ‘10 or more’. The scores were categorised into four groups (0, 1, 2, 3 or more children).

#### Income

Participants were asked ‘Please choose from the following options to indicate your approximate gross (before tax is taken away) household income in 2019 (last year). Include income from partners and other family members living with you and all kinds of earnings including salaries and benefits’ and to choose one of five categories: ‘£0–£300 per week (equals about £0–£1290 per month or £0–15 490 per year)’, ‘£301–£490 per week (equals about £1291–£2110 per month or £15 491–£25 340 per year)’, ‘£491–£740 per week (equals about £2111–£3230 per month or £25 341–£38 740 per year)’, ‘£741–£1111 per week (equals about £3231–£4830 per month or £38 741–£57 930 per year)’ and ‘£1112 or more per week (equals about £4831 or more per month or £57 931 or more per year)’.

#### Loss of income

Participants were asked ‘Some people have lost income because of the coronavirus COVID-19 pandemic, for example because they have not been able to work as much or because business contracts have been cancelled or delayed. Please indicate whether your household has been affected in this way’, and the response options were ‘My household has lost income because of the coronavirus COVID-19 pandemic’, ‘My household has not lost income because of the coronavirus COVID-19 pandemic, and ‘I do not know whether my household has lost income because of the coronavirus COVID-19 pandemic’. The first option was considered as ‘Yes’ (1) and the other options were collapsed to represent ‘No’.

#### Health problems

Participants were asked ‘Do you have diabetes, lung disease, or heart disease?’, and the response options were ‘Yes’ (1) and ‘No’ (0). They were also asked ‘Do any of your immediate family have diabetes, lung disease, or heart disease?’, and the response options were ‘Yes’ (1) and ‘No’ (0).

#### COVID-19 status, self and other

Participants were asked ‘Have you been infected by the coronavirus COVID-19?’, and six responses were provided. These were collapsed into a binary variable representing ‘Perceived infection status’. Positive perceived infection status was based on the selection of either, ‘I have the symptoms of the COVID-19 virus and think I may have been infected’ or ‘I have been infected by the COVID-19 virus and this has been confirmed by a test’. Negative perceived infection status was based on the selection of either, ‘No. I have been tested for COVID-19 and the test was negative’, ‘No, I do not have any symptoms of COVID-19’, ‘I have a few symptoms of cold or flu but I do not think I am infected with the COVID-19 virus’ or ‘I may have previously been infected by COVID-19 but this was not confirmed by a test and I have since recovered’. Positive status (self) was coded ‘1’ and negative status was coded as ‘0’.

Participants were also asked ‘Has someone close to you (a family member or friend) been infected by the coronavirus COVID-19?’, and four responses were provided. These were collapsed into a binary variable representing ‘Perceived infection status – someone close’. Positive perceived infection status was based on the selection of either, ‘Someone close to me has symptoms, and I suspect that person has been infected’ or ‘Someone who is close to me has had a COVID-19 virus infection confirmed by a doctor’. Negative perceived infection status was based on the selection of either, ‘No’ or ‘Someone close to me has symptoms, but I am not sure if that person is infected’. Positive status (other) was coded ‘1’ and negative status was coded as ‘0’.

#### Perceived risk of COVID-19 infection

Participants were asked ‘What do you think is your personal percentage risk of being infected with the COVID-19 virus over the following time periods?’, and three sliders were presented, one for each time period: (1) ‘In the next month’, (2) ‘In the next three months’, (3) ‘In the next six months’? The slider had ‘0’ and ‘100’ at the left- and right-hand extremes, respectively, with 10 point increments, and the labels ‘No Risk’, ‘Moderate Risk’ and ‘Great Risk’ were shown on the left-hand, middle and right-hand parts of the scale, respectively. These produced continuous scores for each time period, ranging from 0 to 100, with higher scores reflecting higher levels of perceived risk of being infected by COVID-19. The scores were recoded into ‘low’ (0–33), ‘moderate’ (34–67) and ‘high’ (68–100).

#### Depression

Nine symptoms of depression were measured using the Patient Health Questionnaire-9 (PHQ-9).^13^ Participants indicated how often they had been bothered by each symptom over the past 2 weeks using a four-point Likert scale ranging from 0 (not at all) to 3 (nearly every day). Possible scores ranged from 0 to 27, with higher scores indicative of higher levels of depression. To identify participants likely to meet the criteria for depressive disorder, a cut-off score of 10 was used. This cut-off produces adequate sensitivity (0.85) and specificity (0.89), corresponds to ‘moderate’ levels of depression^14^ and is used to identify a level of depression that may require psychological intervention.^15^ The psychometric properties of the PHQ-9 scores have been widely supported, and the reliability of the scale among the current sample was excellent (*α* = 0.92).

#### Generalised anxiety

Symptoms of generalised anxiety were measured using the Generalized Anxiety Disorder 7-item Scale (GAD-7).^16^ Participants indicated how often they had been bothered by each symptom over the past 2 weeks on a four-point Likert scale (0 = Not at all, to 3 = Nearly every day). Possible scores ranged from 0 to 21, with higher scores indicative of higher levels of anxiety. A cut-off score of 10 was used; this has been shown to result in sensitivity of 89 % and a specificity of 82 %.^16^ The GAD-7 has been shown to produce reliable and valid scores in community studies,^17^ and the reliability in the current sample was high (*α* = 0.94).

#### Traumatic stress

The International Trauma Questionnaire (ITQ)^18^ is a self-report measure of ICD-11 PTSD based on a total of six symptoms across the three symptom clusters of re-experiencing, avoidance and sense of threat: each symptom cluster comprises two symptoms. Participants were asked to complete the ITQ ‘… in relation to your experience of the COVID-19 pandemic. Please read each item carefully, then select one of the answers to indicate how much you have been bothered by that problem in the past month’. The PTSD symptoms are accompanied by three items measuring functional impairment caused by these symptoms. All items are answered on a five-point Likert scale, ranging from 0 (not at all) to 4 (extremely), with possible scores ranging from 0 to 24. A score of ≥2 (moderately) is considered ‘endorsement’ of that symptom. A PTSD diagnosis requires traumatic exposure and at least one symptom to be endorsed from each PTSD symptom cluster (re-experiencing, avoidance and sense of threat), and endorsement of at least one indicator of functional impairment. The psychometric properties of the ITQ scores have been demonstrated in multiple general populations^19,20^ and in clinical and high-risk samples.^21,22^ The reliability of the PTSD items was high (*α* = 0.93).

#### COVID-19-related anxiety

The survey included a question ‘How anxious are you about the coronavirus COVID-19 pandemic?’, and the participants were provided with a ‘slider’ (electronic visual analogue scale) to indicate their degree of anxiety with ‘0’ and ‘100’ at the left- and right-hand extremes, respectively, and 10 point increments. This produced continuous scores ranging from 0 to 100, with higher scores reflecting higher levels of COVID-19-related anxiety. The scores were recoded into quintiles, and the upper quintile was considered to be indicative of ‘COVID-19 anxiety’.

Similar recruitment strategies and measures have been used by international collaborators in other countries, including Ireland,^10^ Italy, Spain, Saudi Arabia and the United Arab Emirates.

### Analysis plan

The analyses were conducted in three linked phases. First, the prevalences of generalised anxiety, depression and traumatic stress were estimated using the established cut-off scores. Second, the bivariate associations between the predictor variables and the mental health variables were calculated using logistic regression, and the associations were reported as odds ratios (ORs) with 95 % confidence intervals. Third, all predictor variables were entered simultaneously into multivariate binary logistic regression models to estimate the unique effect of each predictor variable, and the associations were reported as ORs.

## Results

Based on the cut-off scores for the GAD-7 and the PHQ-9, the prevalence of depression was 22.1 % (95 % CI 20.3–23.9 %) and that of anxiety was 21.6 % (95 % CI 19.8–23.4 %). There was no significant difference between prevalence of depression for males and females (*χ*^2^ (1) = 2.34, *P* = 0.12), but significantly more females (25.1 %) screened positive for anxiety than males (17.9 %: *χ*^2^ (1) = 15.48, *P* < 0.001). A variable was computed to represent participants who screened positive for the most common mental health disorders (anxiety/depression), either anxiety or depression; the prevalence for this was 27.7 % (95 % CI 25.8–29.7 %), and the prevalence was higher for females (31.7 %) than for males (23.4 %: (*χ*^2^ (1) = 17.57, *P* < 0.001). Using the diagnostic algorithm for the ITQ, the prevalence of traumatic stress was 16.79 % (95 % CI 15.2–18.4 %). There was a significant gender difference, with a higher prevalence of traumatic stress for males (18.9 %) compared with females (14.9 %: *χ*^2^ (1) = 5.85, *P* < 0.01). The COVID-19 anxiety prevalence was 21.3 % (95 % CI 19.5–23.1 %), and there was a significant gender difference, with a higher prevalence of COVID-19 anxiety for females (24.6 %) compared with males (17.7 %: *χ*^2^ (1) = 5.85, *P* < 0.01).

Three binary logistic regression models were used to predict caseness on COVID-19-related anxiety, anxiety/depression and traumatic stress. The predictor variables were age, gender, living location, lone adult status, number of children, income, loss of income, pre-existing health condition (self and other), COVID-19 infection status (self and other) and personal risk of infection over the following month.

Table 1 shows the findings for COVID-19-related anxiety, stratified by the predictor variables, with bivariate associations (unadjusted) presented as ORs, and ORs from the multivariate (adjusted) model with all predictors entered. The multivariate model was significant (*χ*^2^ (24) = 139.97, *P* < 0.001). When the unadjusted ORs were calculated, only female gender, the presence of children in the household and estimates of personal risks of infection were predictive of COVID-related anxiety. However, when the adjusted effects were calculated, the effect for the presence of children became stronger; there was an effect for history of infection, which should be interpreted with caution in the light of the small numbers involved; and there was a very strong effect for age, with older participants reporting more anxiety about the virus.

**Table 1:** Bivariate and multivariate binary logistic regression results predicting COVID-related anxiety

		COVID-19 anxiety	Unadjusted OR	Adjusted OR
	*N*	*N* (%)		
Age				
18–24	246	42 (17.1 %)	–	–
25–34	380	66 (17.4 %)	1.02 (0.667–1.56)	0.93 (0.59–1.46)
35–44	353	75 (21.2 %)	1.31 (0.86–1.99)	1.40 (0.88–2.21)
45–54	410	96 (23.4 %)	1.48 (0.99–2.22)	1.99 (1.28–3.07)**
55–64	349	84 (24.1 %)	1.54 (1.02–2.33)*	2.58 (1.63–4.08)***
65+	287	68 (23.7 %)	1.51 (0.98–2.32)	2.42 (1.50–3.91)***
Gender				
Female	1047	258 (24.6 %)	–	–
Male	972	172 (17.7 %)	0.65 (0.53–0.82)***	0.586 (0.463–0.743)***
Living location				
Rural	335	74 (22.1 %)	–	–
Town	620	130 (21.0 %)	0.94 (0.68–1.29)	0.92 (0.65–1.29)
Suburb	572	106 (18.5 %)	0.80 (0.57–1.12)	0.77 (0.54–1.09)
City	498	121 (24.3 %)	1.13 (0.81–1.57)	1.20 (0.84–1.71)
Lone adult				
No	1571	337 (21.5 %)	–	–
Yes	454	94 (20.7 %)	0.96 (0.740–1.24)	0.971 (0.716–1.317)
Children				
0	1429	283 (19.7 %)	–	–
1	292	56 (19.1 %)	0.96 (0.70–1.32)	1.09 (0.77–1.55)
2	237	73 (30.7 %)	1.80 (1.33–2.44)***	2.11 (1.49–2.98)***
3+	61	19 (31.1 %)	1.84 (1.05–3.21)*	2.35 (1.29–4.28)**
Income				
£57 930+	410	77 (18.8 %)	–	–
−£57 930 pa	410	86 (21.0 %)	1.15 (0.81–1.62)	1.15 (0.80–1.65)
−£38 740 pa	385	88 (22.9 %)	1.28 (0.91–1.81)	1.40 (0.97–2.03)
−£25 340 pa	410	86 (21.0 %)	1.15 (0.82–1.62)	1.37 (0.94–2.02)
£0–15 490 pa	410	94 (22.9 %)	1.29 (0.92–1.80)	1.30 (0.881–1.92)
Lost income				
Not lost	1377	282 (20.5 %)	–	–
Lost	648	149 (23.0 %)	1.16 (0.93–1.45)	1.18 (0.93–1.51)
Pre-existing health condition, self				
No	1714	348 (20.3 %)	–	–
Yes	311	83 (26.7 %)	1.43 (1.08–1.89)*	1.24 (0.91–1.69)
Pre-existing health condition, someone close				
No	1510	305 (20.2 %)	–	–
Yes	515	126 (24.5 %)	1.28 (1.01–1.62)*	1.07 (0.82–1.39)
COVID-19, self				
No	1977	425 (21.5 %)	–	
Yes	48	6 (12.5 %)	0.52 (0.22–1.23)	0.39 (0.16–.99)*
COVID-19, someone close				
No	1913	407 (21.3 %)	–	–
Yes	112	24 (21.4 %)	1.01 (0.63–1.61)	0.89 (0.54–1.45)
Personal risk 1 month				
Low	633	81 (12.8 %)	–	
Moderate	867	182 (21.0 %)	1.81 (1.36–2.41)***	1.75 (1.31–2.34)***
High	525	168 (32.0 %)	3.21 (2.38–4.31)***	3.14 (2.31–4.28)***

* *P* < 0.05, **P < 0.01, ***P < 0.001.

The multivariate regression models for both anxiety/depression (*χ*^2^ (24) = 292.03, *P* < 0.001), and traumatic stress (*χ*^2^ (24) = 328.58, *P* < 0.001) were statistically significant; the unadjusted and adjusted ORs are shown in Tables 2 and 3. For anxiety/depression, there was a strong effect for age, contrary to the effect observed for COVID-related anxiety, with very high levels of psychological symptoms in the youngest participants and low levels in those over 65 years of age. A bivariate effect for urban location did not survive in the multivariate model, and the effect of having children in the house was much muted in the multivariate model. Participants who had lost income in the pandemic and those in the lower-income categories showed markedly higher risk for anxiety/depression. Higher levels of anxiety/depression were also reported by those who had pre-existing health conditions, knew someone who had a pre-existing health condition, had become infected themselves, and/or gave a high estimate of their personal risk of infection.

**Table 2:** Bivariate and multivariate binary logistic regression results predicting anxiety/depression

		Anxiety/depression	Unadjusted OR	Adjusted OR
	*N*	*N* (%)		
Age				
18–24	246	121 (49.2 %)	–	–
25–34	380	152 (40.0 %)	0.69 (0.50–0.95)*	0.67 (0.47–0.95)*
35–44	353	97 (27.5 %)	0.39 (0.278–0.551)***	0.408 (0.28–0.60)***
45–54	410	96 (23.4 %)	0.32 (0.22–0.44) ***	0.36 (0.25–0.52)***
55–64	349	68 (19.5 %)	0.25 (0.17–0.36) ***	0.31 (0.21–0.47)***
65+	287	28 (9.8 %)	0.11 (0.07–0.18) ***	0.141 (0.09–0.23)***
Gender				
Female	1047	227 (23.4 %)	–	–
Male	972	332 (31.70 %)	0.65 (0.54-0.80)***	0.89 (0.71–1.12)
Living location				
Rural	335	77 (23.0 %)	–	–
Town	620	167 (26.9 %)	1.23 (0.91–1.68)	1.02 (0.73–1.43)
Suburb	572	138 (24.1 %)	1.06 (0.77–1.46)	0.98 (0.70–1.39)
City	498	180 (36.1 %)	1.90 (1.39–2.59)***	1.21 (0.86–1.7)
Lone adult				
No	1571	424 (27.0 %)	–	–
Yes	454	138 (30.4 %)	1.18 (0.94–1.48)	1.32 (0.99–1.75)
Children				
0	1429	355 (24.8 %)	–	–
1	292	95 (32.4 %)	1.46 (1.11–1.91)**	1.19 (0.88–1.61)
2	237	90 (37.8 %)	1.84 (1.38–2.46)***	1.41 (1.01–1.96)*
3+	61	22 (36.1 %)	1.71 (1.00–2.93)*	1.41 (0.79–2.53)
Income				
£57 930+	410	70 (17.1 %)	–	–
−£57 930 pa	410	91 (22.2 %)	1.39 (0.98–1.96)	1.28 (0.89–1.85)
−£38 740 pa	385	117 (30.4 %)	2.12 (1.51–2.97)***	1.69 (1.17–2.44)**
−£25 340 pa	410	135 (32.9 %)	2.38 (1.71–3.31)***	1.67 (1.15–2.42)**
£0–15 490 pa	410	149 (36.3 %)	2.77 (2.00–3.84)***	2.44 (1.67–3.56)***
Lost income				
Not lost	1377	323 (23.5 %)	–	–
Lost	648	239 (36.9 %)	1.91 (1.56–2.33)***	1.25 (1.25–1.95)***
Pre-existing health condition, self				
No	1714	452 (26.4 %)	–	–
Yes	311	110 (35.4 %)	1.53 (1.18–1.97)**	1.45 (1.07–1.96)*
Pre-existing health condition, someone close				
No	1510	386 (25.6 %)	–	–
Yes	515	176 (34.2 %)	1.51 (1.22–1.88)***	1.33 (1.03–1.74)*
COVID-19, self				
No	1977	535 (27.1 %)	–	
Yes	48	27 (56.3 %)	3.46 (1.94–6.18)***	2.17 (1.14–4.11)**
COVID-19, someone close				
No	1913	515 (26.9 %)	–	–
Yes	112	47 (42.0 %)	1.96 (1.33–2.89)**	1.50 (0.97–2.32)
Personal risk, 1 month				
Low	633	139 (22.0 %)	–	–
Moderate	867	208 (24.0 %)	1.12 (0.88–1.43)	1.13 (0.87–1.47)
High	525	215 (41.0 %)	2.46 (1.91–3.18)***	2.20 (1.66–2.91)***

* *P* < 0.05, ** *P* < 0.01, ***P < 0.001.

**Table 3:** Bivariate and multivariate binary logistic regression results predicting traumatic stress

		Traumatic stress	Unadjusted OR	Adjusted OR
	*N*	*N* (%)		
Age				
18–24	246	59 (24.0 %)	–	–
25–34	380	109 (28.7 %)	1.27 (0.88–1.84)	0.99 (0.65–1.49)
35–44	353	88 (24.9 %)	1.05 (0.72–1.54)	0.74 (0.48–1.15)
45–54	410	53 (12.9 %)	0.47 (0.31–0.71)***	0.39 (0.25–0.62)***
55–64	349	24 (6.9 %)	0.23 (0.14–0.39)***	0.31 (0.18–0.54)***
65+	287	7 (2.4 %)	0.08 (0.03–0.18)***	0.09 (0.04–0.22)***
Gender				
Female	1047	156 (14.9 %)	–	–
Male	972	184 (18.9 %)	1.33 (1.06–1.68)*	1.85 (1.41–2.44) ***
Living location				
Rural	335	36 (10.7 %)	–	–
Town	620	76 (12.3 %)	1.16 (0.76–1.77)	0.94 (0.60–1.50)
Suburb	572	88 (15.4 %)	1.51 (0.99–2.28)	1.24 (0.79–1.94)
City	498	140 (28.1 %)	3.25 (2.18–4.83)***	1.91 (1.23–2.94)**
Lone adult				
No	1571	268 (17.1 %)	–	–
Yes	454	72 (15.9 %)	0.92 (0.69–1.22)	1.412 (0.99–2.00)
Children				
0	1429	163 (11.4 %)	–	–
1	292	75 (25.6 %)	2.68 (1.96–3.65)***	1.83 (1.30–2.58)**
2	237	83 (34.9 %)	4.17 (3.05–5.70)***	2.56 (1.78–3.68)***
3+	61	19 (31.1 %)	3.52 (2.00–6.21)***	2.39 (1.29–4.44)**
Income				
£57 930+	410	49 (12.0 %)	–	–
−£57 930 pa	410	59 (14.4 %)	1.24–(0.82–1.86)	1.27 (0.82–1.98)
−£38 740 pa	385	81 (21.0 %)	1.96 (1.33–2.88)**	1.55 (0.99–2.40)
−£25 340 pa	410	98 (23.9 %)	2.31 (1.59–3.36)***	1.85 (1.19–2.87)**
£0–15 490 pa	410	53 (12.9 %)	1.09 (0.72–1.65)	1.28 (0.78–2.07)
Lost income				
Not lost	1377	196 (14.2 %)	–	–
Lost	648	144 (22.2 %)	1.722 (1.36–2.18)***	1.27 (0.97–1.66)
Pre-existing health condition, self				
No	1714	279 (16.3 %)	–	–
Yes	311	61 (19.6 %)	1.25 (0.92–1.71)	1.21 (0.829–1.77)
Pre-existing health condition, someone close				
No	1510	247 (16.4 %)	–	–
Yes	515	93 (18.1 %)	1.13 (0.87–1.46)	1.13 (0.82–1.56)
COVID-19, self				
No	1977	324 (16.4 %)	–	–
Yes	48	16 (33.3 %)	2.55 (1.38–4.70)**	1.03 (0.50–2.12)
COVID-19, someone close				
No	1913	305 (15.9 %)	–	–
Yes	112	35 (31.3 %)	2.39 (1.57–3.64)***	1.70 (1.04–2.77)*
Personal risk, 1 month				
Low	633	54 (8.5 %)	–	–
Moderate	867	132 (15.2 %)	1.92 (1.37–2.69)***	1.88 (1.32–2.68)**
High	525	154 (29.3 %)	4.45 (3.18–6.23)***	3.55 (2.47–5.09)***

* *P* < 0.05, **P < 0.01, ***P < 0.001.

Finally, in the case of traumatic stress, there was again a higher prevalence in younger participants, but the gender effect was reversed compared with anxiety/depression, with more symptoms being reported by males. The influence of the presence of children was marked for both the bivariate associations and the multivariate model, but there was little effect for income or loss of income when other variables were controlled for. The lack of an association for being infected by COVID-19 in the multivariate model should be interpreted with caution, given the small numbers involved and the wide confidence intervals. Trauma symptoms were also associated with the perception of a high risk of infection.

## Discussion

This study was one of the first to measure psychological disorders in a representative sample of the UK population during a pandemic. The study had the additional virtues of recruiting participants early in the crisis and using standardised measures, allowing follow-up at later stages. We found higher levels of anxiety, depression and traumatic stress than those previously reported by general population-based studies. Although previous studies have investigated the psychological effects of past pandemics, particularly the SARS and H1N1 pandemics in the far east, they mostly considered the effects on pandemic survivors and health professionals, and the only population-based studies did not use standardised instruments. For example, a study in Taiwan following the 2003 SARS pandemic used a five-item symptom-rating scale, and found that poorer mental health was related to personal experience of SARS or knowing people who had been affected.^11^ In a Chinese study that employed a short questionnaire during the same pandemic, respondents reported increased fear, anxiety and panic.^2^ However, a longitudinal study of citizens of Hong Kong during the 2009 H1N1 pandemic found low levels of anxiety throughout, but anxiety levels were associated with compliance with social distancing advice.^1^

Our primary aim was to assess the levels of anxiety, depression and traumatic stress in the population during the early stages of the COVID-19 pandemic. The prevalence of anxiety (21.63 %) and depression (22.12 %) found in this study appear to be higher than those previously reported, but not markedly so. The English 2014 Adult Psychiatric Morbidity Survey (APMS)^23^ reported that 15.7 % of the sample experienced symptoms of common mental health disorders, based on a cut-off score of 12 on the Clinical Interview Schedule-Revised, with a higher prevalence for women (19.1 %) than for men (12.2 %). The prevalence of anxiety or depression in the Understanding Society study in 2014 was 19.7 % (22.5 % for females, 16.8 % for males),^24^ based on the General Health Questionnaire (GHQ). The closest comparable study is probably the National Institute for Health Research Applied Research Collaboration North West Coast Household Health Survey, which administered the PHQ9 and GAD7 (face-to-face) to 4000 people in the north-west of England, mainly living in deprived areas; in this study, 17 % were depressed and 13 % were anxious.^25^ A recently published study used data from the Understanding Society COVID-19 web survey, and reported the population prevalence of clinically significant levels of mental distress to be 27.3 %.^26^ The study used the GHQ to identify clinically significant distress, and data collection was approximately 1 month after our data collection period, but despite these differences the GHQ prevalence was similar to that based on meeting the criteria for either anxiety or depression in this study, which was 27.7 %. This may be indicative of a stable psychological response during the first month of lockdown, although longitudinal studies will be required to determine the longitudinal change during lockdown.

The prevalence of PTSD in this current study was 16.79 %, similar to the combined prevalence of PTSD and complex PTSD in a UK trauma-exposed sample (prevalence of 5.3 % for PTSD and 12.9 % for complex PTSD^27^), and much higher than that reported by the APMS (4.4 %, with no gender differences found^11^). However, these comparisons should be treated with caution, as the status of COVID-19 as a traumatic stressor is not clear. Unexpectedly, the prevalence for males was higher than that for females; most epidemiological studies report a higher prevalence of PTSD for females.^28^ The reasons for this are not immediately clear, but the health and economic threats that COVID-19 poses may be undermining traditional male gender roles, or the higher prevalence of mortality for males during the British COVID-19 pandemic may play a part.

The unadjusted estimates for the model predicting anxiety/depression revealed that younger age, being female, living in a city, pre-existing health conditions, COVID-19 status and perceived risk of COVID-19 infection all significantly increased the likelihood of screening positive for anxiety or depression.

Contrary to expectations, the oldest age group and being male were associated with a lower likelihood of anxiety or depression, despite these factors being associated with higher COVID-19-related mortality.^29^ In the 2014 Adult Psychiatric Morbidity Survey, a much lower prevalence of common psychological disorders was observed in those over 65 compared with those of working age, although the effect was nonlinear and the high prevalence observed for those under 35 in this study were not evident there. Strikingly, the opposite relationship with age was observed for anxiety specifically about the COVID-19 pandemic, which was related to mortality risk in a logical way. The adjusted estimates were generally attenuated, but the same pattern of associations was found. The unadjusted estimates for the model predicting traumatic stress differed in that being male was a significant risk factor, and there was a large effect for living in an urban area.

This study had both strengths and limitations. On the strengths side, the sample was highly representative of the UK population, was recruited early in the progress of the pandemic, and used standardised measures, allowing comparisons with findings from later stages of the COVID-19 crisis. However, despite the sampling frame and large sample size, and although the participants in this study were representative of the UK population in terms of demographic, economic and social factors, as well as voting history, it was not a true random probability sample (which would have been very difficult to obtain under the current circumstances), and it is possible that individuals’ decisions about whether to participate were affected by psychological factors, creating the possibility of sampling bias. Second, all mental health assessments were based on self-report and not clinician-administered interviews; this may have resulted in overestimation of prevalence. Third, the validity of the assessment of traumatic stress may be questioned, as it is not clear whether the COVID-19 pandemic meets the ICD-11 criteria (‘an extremely threatening or horrific event or series of events’) or DSM-5 criteria (direct exposure, witnessing the trauma, learning that a relative or close friend was exposed to a trauma, indirect exposure to aversive details of the trauma, usually in the course of professional duties) for a traumatic event for the entire population. This question is already being debated,^30^ with arguments being made that the global nature of the threat, its wide ranging effects (i.e. health, economic and social), and the widespread reports of behaviours and cognitions analogous to PTSD symptoms (heightened perceptions of threat, voluntary (and enforced) avoidance, and re-experiencing being facilitated by mainstream and social media) mean that the pandemic should be considered a traumatic stressor. Finally, the mechanisms by which the threat of the pandemic and/or the quarantine influenced mental health could not be established. Previous research has identified disruptions in circadian rhythms,^31^ disruptions in social contact^32^ and quarantine related stressors as important contributing factors.^33^

### Conclusions

Modelling studies have suggested that the influence of pandemics on psychological disorders in the general population may affect the progress of a pandemic and, therefore, indirectly affect mortality.^6^ Furthermore, the development of psychological disorders in the population may create a burden that impedes national social and economic recovery once the pandemic ends. The fact that the prevalence of psychological problems observed in the present study was not dramatically higher than those reported in previous studies suggests that the population, at an early stage of the pandemic, has successfully adapted to the unprecedented changes that have been forced on their lifestyles. However, we have identified certain key groups who may be more vulnerable to the social and economic challenges of the pandemic, particularly those whose income has been affected, who have children living in the home and who have pre-existing health conditions that make them vulnerable to the more devastating effects of the COVID-19 virus. Further research is needed to track whether these groups show higher levels of psychological problems at later stages in the pandemic and whether specific interventions and policies should be developed to address their needs.

## Data Availability

The datasets generated during and/or analysed during the current study will be archived with the UK Data Service (https://ukdataservice.ac.uk/) within 6 months of the study ending.
